# The relationship of serum vitamins A, D, E and LL-37 levels with allergic status, tonsillar virus detection and immune response

**DOI:** 10.1371/journal.pone.0172350

**Published:** 2017-02-24

**Authors:** Varpu Elenius, Oscar Palomares, Matti Waris, Riitta Turunen, Tuomo Puhakka, Beate Rückert, Tytti Vuorinen, Tobias Allander, Tero Vahlberg, Mübeccel Akdis, Carlos A. Camargo, Cezmi A. Akdis, Tuomas Jartti

**Affiliations:** 1 Department of Pediatrics, Turku University Hospital, Turku, Finland; 2 Swiss Institute of Allergy and Asthma Research (SIAF), University of Zürich, Christine Kühne-Center for Allergy Research and Education (CK-CARE), Davos, Switzerland; 3 Department of Biochemistry and Molecular Biology, School of Chemistry, Complutense University of Madrid (UCM), Madrid, Spain; 4 Department of Virology, University of Turku, Turku, Finland; 5 Department of Otorhinolaryngology, Turku University Hospital, Turku, Finland; 6 Department of Otorhinolaryngology, Satakunta Central Hospital, Pori, Finland; 7 Department of Clinical Microbiology, Karolinska University Hospital, Stockholm, Sweden; 8 Department of Biostatistics, University of Turku, Turku, Finland; 9 Department of Emergency Medicine, Massachusetts General Hospital, Harvard Medical School, Boston, United States of America; National Taiwan University College of Public Health, TAIWAN

## Abstract

**Background:**

Tonsils have an active role in immune defence and inducing and maintaining tolerance to allergens. Vitamins A, D, and E, and antimicrobial peptide LL-37 may have immunomodulatory effects. We studied how their serum levels were associated with allergy status, intratonsillar/nasopharyngeal virus detection and intratonsillar expression of T cell- and innate immune response-specific cytokines, transcription factors and type I/II/III interferons in patients undergoing tonsillectomy.

**Methods:**

110 elective tonsillectomy patients participated. Serum levels of vitamins A, 25(OH)D, and E, LL-37 and allergen-specific IgE as well as nasopharyngeal/intratonsillar respiratory viruses were analyzed. The mRNA expression of IFN-α, IFN-β, IFN-γ, IL-10, IL-13, IL-17, IL-28, IL-29, IL-37, TGF-β, FOXP3, GATA3, RORC2 and Tbet in tonsils were analyzed by quantitative RT-PCR.

**Results:**

The median age of the patients was 16 years (range 3–60), 28% of subjects had atopy, and 57% carried ≥1 respiratory virus in nasopharynx. Detection of viruses decreased by age. Higher vitamin A levels showed borderline significance with less viral detection (*P* = 0.056). Higher 25(OH)D was associated with less allergic rhinitis and atopy (*P* < 0.05) and higher vitamin E with less self-reported allergy (*P* < 0.05). In gene expression analyses, 25(OH)D was associated with higher IL-37, vitamin A with higher IFN-γ and vitamin E with less IL-28 (*P* < 0.05). LL-37 was associated with less FOXP3, RORC2 and IL-17 in tonsils (*P* < 0.05).

**Conclusions:**

Vitamin D and E levels were associated with less allergic disorders. Vitamin A was linked to antiviral and vitamin D with anti-inflammatory activity. LL-37 and was linked to T regulatory cell effects.

## Introduction

Epidemiologic and multiple observational studies suggest that deficiencies of vitamins A, D and E may be associated with development of asthma and allergic disorders [1–4]. It was found in several studies that vitamin A deficiency is associated with a higher risk of asthma [5–7], but randomized trials with vitamin A supplementation were less supportive [8, 9]. Prospective studies have shown that vitamin D supplementation reduces the risk of recurrent respiratory infections, virus-induced wheezing and asthma exacerbations although some of the studies have shown conflicting results [10–14]. Vitamin D is known to induce antimicrobial peptide LL-37, which has anti-viral, -bacterial and -fungal effects [15]. Maternal vitamin E intake during pregnancy has been negatively associated with wheezing and eczema in children of atopic mothers [16, 17].

We determined serum levels of vitamins A, D, and E and antimicrobial peptide LL-37 in patients undergoing tonsillectomy. Tonsils are the first contact point of the immune system to various infectious agents, food and aeroallergens [18] and they have an active role in inducing and maintaining tolerance to various allergens [19]. However, it is not known how they regulate these functions. We studied how serum vitamins and antimicrobial peptide LL-37 levels and allergic and tonsillar diseases were associated with direct *in vivo* detection of respiratory viruses and T cell subset-related transcription factors, cytokines as well as type I, II and III interferons in tonsils.

## Methods

### Patients

Human tonsil samples were obtained from 110 elective tonsillectomy patients (Table 1) from Satakunta Central Hospital, Pori, Finland, from April 2008 to March 2009 and biobanked. Tonsillectomy was done according to clinical indications. Written informed consent was obtained from the study patients and/or their guardians. The ethics committee of Turku University Hospital approved the study. All patients filled a standard questionnaire to obtain information of their allergic diseases and respiratory symptoms. Atopy was defined as positive immunoglobulin E (IgE) antibody (>0.35 kU/L) to any of the following allergens: codfish, cow’s milk, egg, peanut, soybean, wheat, cat, dog, horse, birch, mugwort, timothy, *Cladosporium herbarum* or *Dermatophagoides pteronyssinus* (Phadiatop Combi^®^, Phadia, Uppsala, Sweden). Animal sensitization was defined as positive IgE antibodies to cat, dog, horse or *Dermatophagoides pteronyssinus*. Birch, mugwort, timothy and *Cladosporium herbarum* were considered as pollen aeroallergens. The eczema was defined as atopic eczema, if a child was atopic and had typical symptoms that included pruritus, typical morphology and chronicity of atopic eczema (Hanifin and Rajka Diagnostic Criteria for Atopic Dermatitis).

**Table 1:** The patient characteristics.

Factor	n = 110
Age (years)^1^	16 (8, 27)
Male	50 (45%)
Indication for tonsillectomy	
Hypertrophic tonsils	47 (43%)
Recurrent tonsillitis	42 (38%)
Other indication	6 (5%)
Mixed indications of these	15 (14%)
Self-reported allergy	50/99 (51%)
Atopy	24/87 (28%)
Food	12/87 (14%)
Pollen, animal or house dust mite	12/87 (14%)
Physician-diagnosed allergic rhinitis	27/100 (27%)
Physician-diagnosed atopic eczema	14/102 (14%)
Physician-diagnosed asthma	12/99 (12%)
Active smoking	19/98 (19%)
Respiratory symptoms on the operation day^2^	16/95 (17%)
Last day of respiratory symptoms, days ago^3^	20 (7, 31)
Respiratory symptoms within 2 week	37/96 (39%)
Respiratory symptoms within 4 weeks	50/96 (52%)

Values are shown as medians (interquartile range) or n (%).

^1^Range 3 to 60 years.

^2^Four had throat symptoms, two had rhinitis and two had cough, one had symptoms of upper airway obstruction and 5 had combinations of these.

^3^If >30 days, 31 days was used in the calculation. Throat symptoms were excluded from the calculation.

### Sample collection

Serum samples were taken before surgery. Tonsillectomy was performed according to clinical routine. Tonsil tissue was immediately cut in 3–4 mm cubes in sterile conditions, stored in RNA*late*r RNA stabilization reagent (Qiagen, Hilden, Germany), incubated at 2–8°C until the next working day and stored in -80°C after removal of the non-absorbed reagent [20]. The nasopharyngeal aspirate samples were obtained during the operation using a standardized procedure as previously described [21]. Both nasopharyngeal aspirate and sera were stored in -80°C before analyses.

### Sample analysis

Retinoic acid (vitamin A) and alpha tocopherol (vitamin E) levels were determined by high-performance liquid chromatography (HPLC) in the Vita Laboratory, Helsinki, Finland. Serum total 25(OH)D measurement was done using an immunoassay (Abbott Architect, Chicago, USA) and LL-37 was measured using ELISA (Hycult Biotech, Uden, the Netherlands), both in Massachusetts General Hospital, Boston, USA. Bioavailable levels of 25(OH)D were estimated using additional serum measurements (D-binding protein and albumin) and published formulae [22]. Serum specific IgE levels against common airborne and food allergens were determined by using a fluoroenzyme immunoassay (cut-off for specific allergens 0.35 kU/l; ImmunoCAP, Phadia, Uppsala, Sweden) in Turku University Hospital, Turku, Finland.

Viral diagnostics of naive nasopharyngeal aspirates and intratonsillar samples were performed according to clinical routine using PCRs for adenovirus, bocavirus-1, coronaviruses (229E, OC43, NL63 and HKU1), enteroviruses, influenza A and B viruses, metapneumovirus, parainfluenza virus types 1–4, respiratory syncytial virus and rhinovirus (including species A, B and C) for all samples, and polyomaviruses KI and WU for 110 samples in the Department of Virology, University of Turku, Turku, Finland and in the Department of Clinical Microbiology, Karolinska University Hospital, Stockholm, Sweden [20].

A nasopharyngeal aspirate sample was suspended into 1 ml of PBS, and nucleic acid was isolated from 550 μl of the suspension using NucliSense easyMag automated nucleic acid extractor (BioMerieux, Boxtel, The Netherlands) with on-board lysis. Intratonsillar samples (approximately 300 μg each) were homogenized and the total RNA was isolated from tonsil tissues as previously described [20]. Reverse transcription was performed with the Revert Aid M-MuLV Reverse Transcriptase (Fermentas, St. Leon-Rot, Germany) using random hexamer primers according to the manufacturers protocol. We analyzed intratonsillar mRNA expression levels of the cytokines and transcription factors related to T subsets cells relevant to allergic responses as well as type I/III interferons related to antiviral responses (Table 2). Gene expressions of IFN-α, IFN-β, IFN-γ, IL-10, IL-13, IL-17, IL-28, IL-29, IL-37, TGF-β, FOXP3, GATA3, RORC2 and Tbet were analyzed by quantitative real-time as previously described [20]. Elongation factor 1α (EF1α) was used as a housekeeping gene. Data are shown as relative expressions, which show 2^-(ΔCT)^ values multiplied by 10^4^, where ΔCT corresponds to the difference between the CT value for the gene of interest and EF1α.

**Table 2:** Intratonsillar transcription factor and cytokine expressions.

Factor	Relative expression n = 110
**T-helper**_**1**_	
Tbet	51 (29, 75)
IFN-γ	64 (35, 110)
**T-helper**_**2**_	
GATA3	24 (16, 40)
IL-13	0.62 (0.026, 3.5)
**T-helper**_**17**_	
RORC2	21 (11, 33)
IL-17	11 (6.0, 19)
**T-regulatory**	
IL-10	46 (26, 70)
TGF-β	170 (110, 220)
FOXP3	49 (28, 87)
IL-37	0.19 (0.12, 0.34)
**Type I/III interferons**	
IFN-α	12 (0.37, 59)
IFN-β	23 (3.0, 110)
IL-28	23 (1.8, 79)
IL-29	7.6 (1.5, 33)

Cytokine and transcription factor data are shown as relative expression, which represents the 2-(ΔCT) values multiplied by 10^4^, where ΔCT corresponds to the difference between the CT value for the gene of interest and the housekeeping gene EF1α. Statistics are shown as means (sd) or medians (interquartile range).

### Statistical analysis

Continuous variables were described as means (SDs) or medians (interquartile ranges) when appropriate, and categorical variables as frequencies and percentages. The subjects with and without serum samples were compared using Mann-Whitney U-test and chi-square test. Correlations were calculated using Spearman rank-order correlations coefficients due to mainly skewed distributions. The associations of serum levels of vitamins and LL-37, allergy status and virus detection with intratonsillar cytokine and transcription factor expressions were analyzed using univariable and age-adjusted linear regression. The modifying effects of age (<16 vs. ≥16 years) and indication of tonsillectomy (recurrent tonsillitis vs. hypertrophic tonsils) on the associations were also examined. Analyses were also adjusted for smoking. Before analyses, vitamin D and LL-37 levels and gene expression values were log transformed because of positively skewed distributions. Statistical significance was established at the level of *P* < 0.05. Statistical analyses were done using SAS System for Windows (Version 9.4, SAS Institute Inc. Cary, NC, USA).

## Results

### Study cohort

Initially, tonsil samples were available from 143 patients and analysed for clinical data, nasopharyngeal/intratonsillar virology and intratonsillar gene expression. Serum samples were available from 110 subjects of these, who were included in the study. The subjects without serum samples did not differ from the analytic cohort in regard to age, sex, allergy or nasopharyngeal/intratonsillar virus detection (all *P* > 0.1).

### Patient characteristics

The median age of the study subjects was 16 years (range 3–60) and 45% were males. Main indications for tonsillectomy were hypertrophic tonsils (43%), recurrent tonsillitis (38%), other indications (5%) or a combination of these indications (14%) (Table 1). Altogether, 51% of patients had self-reported allergy and 28% had atopy, 27% had physician-diagnosed allergic rhinitis, 14% physician-diagnosed atopic eczema and 12% physician-diagnosed asthma (Table 1). Seventeen % of patients had respiratory symptoms on the operation day (Table 1).

### Serum levels of vitamins and LL-37

**Fig 1:**
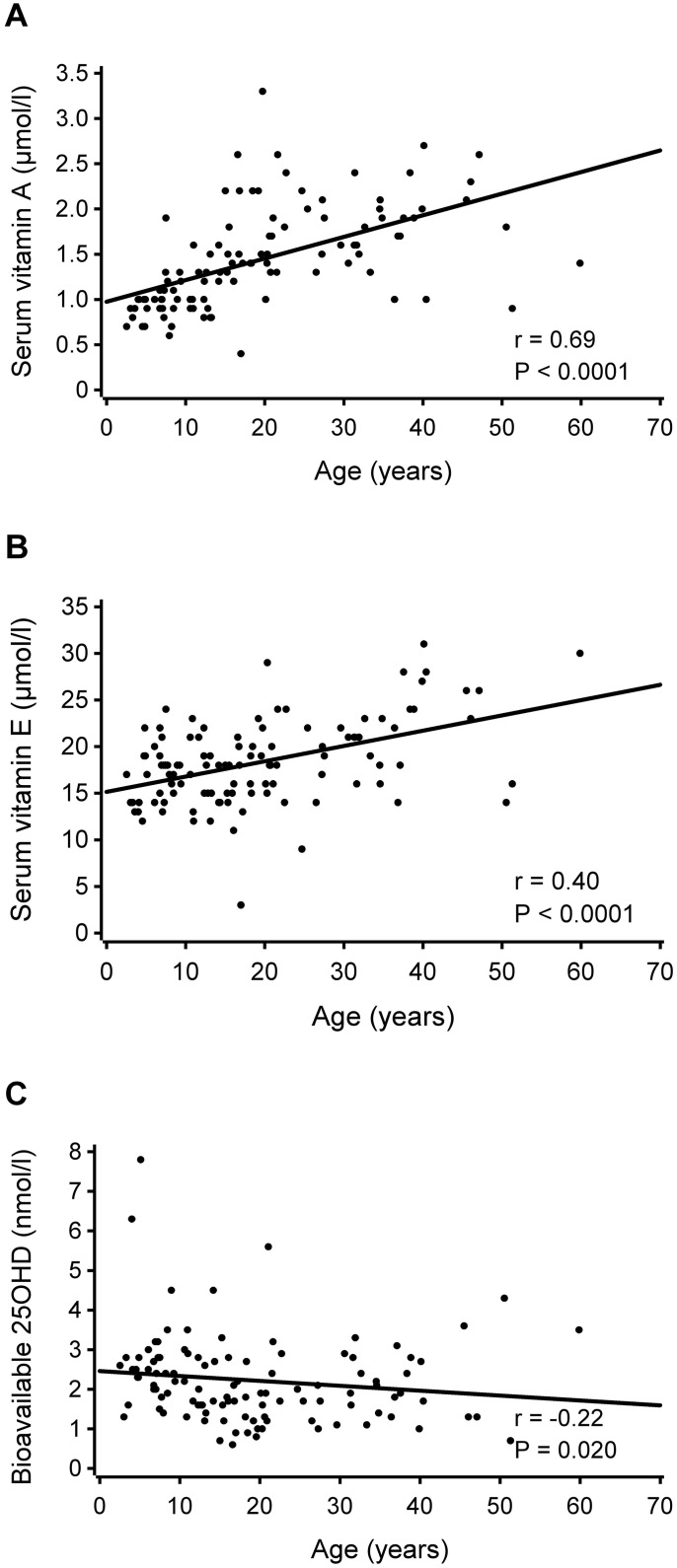
Correlations between age and (A) serum vitamin A, (B) serum vitamin E and (C) serum bioavailable 25(OH)D. Spearman’s correlations are shown. Regression lines have been added for better visualization.

The median level for serum vitamin A was 1.4 μmol/l (range 0.4–3.3), for vitamin E 18 μmol/l (range 3–31), for total 25(OH)D 50 nmol/l (range 15–135), for bioavailable 25(OH)D 2.0 nmol/l (range 0.6–7.8), and for LL-37 34 ng/ml (range 12–525). Both vitamin A and E levels increased by age (*P* <0.0001) (Fig 1A and 1B), but serum bioavailable 25(OH)D levels slightly decreased by age (*P* = 0.02) (Fig 1C). Total 25(OH)D and antimicrobial peptide LL-37 levels did not vary by age (*P* = 0.57) (S1 Fig).

### Virus infections

**Fig 2:**
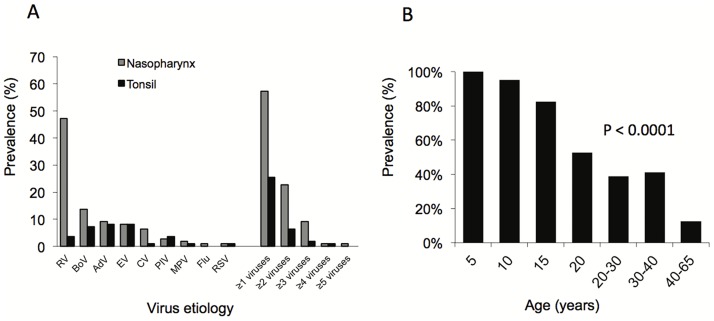
(A) Prevalence of different viruses in nasopharynx and tonsils by PCR. Rhinovirus (RV), bocavirus-1 (BoV), adenovirus (AdV), enteroviruses (EV), coronavirus (CV), parainfluenza virus types 1–4 (PIV), metapneumovirus (MPV), influenza A or B virus (Flu), respiratory syncytial virus (RSV). (B) The prevalence of viruses in nasopharyngeal secretion and/or in tonsils decreases by age.

In the nasopharyngeal aspirates, 57% of the patients had at least one virus and 23% had 2 or more viruses (Fig 2A). Rhinovirus (47%) was the most prevalent virus, followed by bocavirus-1 (14%), adenovirus (9%), enteroviruses (8%), coronavirus (6%) and other viruses (<3% each) (Fig 2A). In tonsils, 25% of patients had at least one virus and 6% had 2 or more viruses (Fig 2A). Bocavirus-1 was detected in 7%, adenovirus and enteroviruses in 8%, and parainfluenza and rhinovirus in 4% of the tonsils (Fig 2A). Virus detection rates strongly decreased by age (both *P* < 0.0001) (Fig 2B). Overall virus prevalence was 100% in children under age 5 years, but only 13% after 40 years of age (Fig 2B).

### Clinical associations

**Fig 3:**
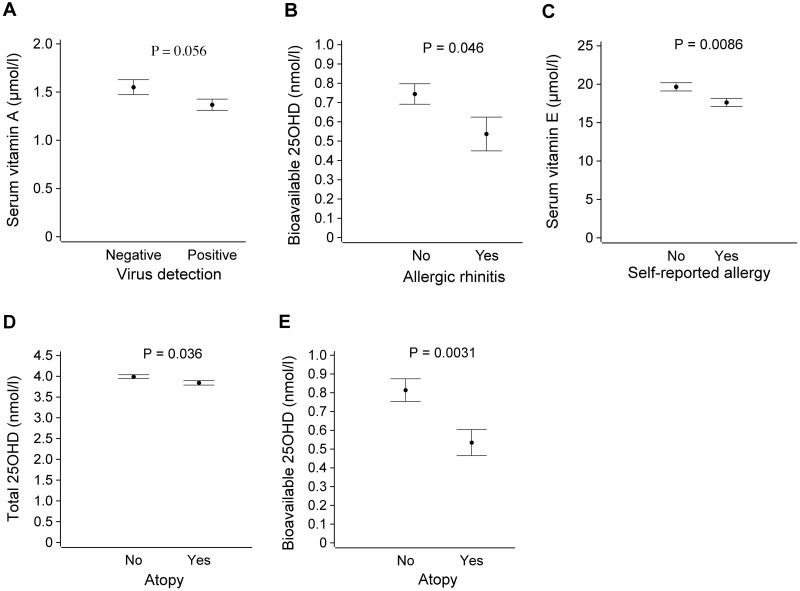
(A) Serum vitamin A levels according to virus detection in nasopharynx and/or tonsils (p = 0.056). (B) Serum bioavailable 25(OH)D levels according to allergic rhinitis status (p = 0.046). (C) Serum vitamin E levels according to self reported allergy (p = 0.0086). (D) Serum total 25(OH)D levels according to atopy (p = 0.036). (E) Serum bioavailable 25(OH)D levels according to atopy (p = 0.0031). Means, standard error of means and adjusted P values are shown.

In age-adjusted analyses, higher vitamin A tended to associate with less nasopharyngeal virus detection (*P* = 0.056) (Fig 3A). Lower bioavailable 25(OH)D levels were associated with allergic rhinitis (*P* = 0.046) (Fig 3B) and lower vitamin E levels with self-reported allergy (*P* = 0.0086) (Fig 3C). Lower total (*P* = 0.036) and bioavailable 25(OH)D (*P* = 0.0031) levels were associated with atopy (Fig 3D and 3E, respectively). No other significant associations were found. Age or indication for tonsillectomy did not effect on these clinical associations.

### mRNA expression associations

**Fig 4:**
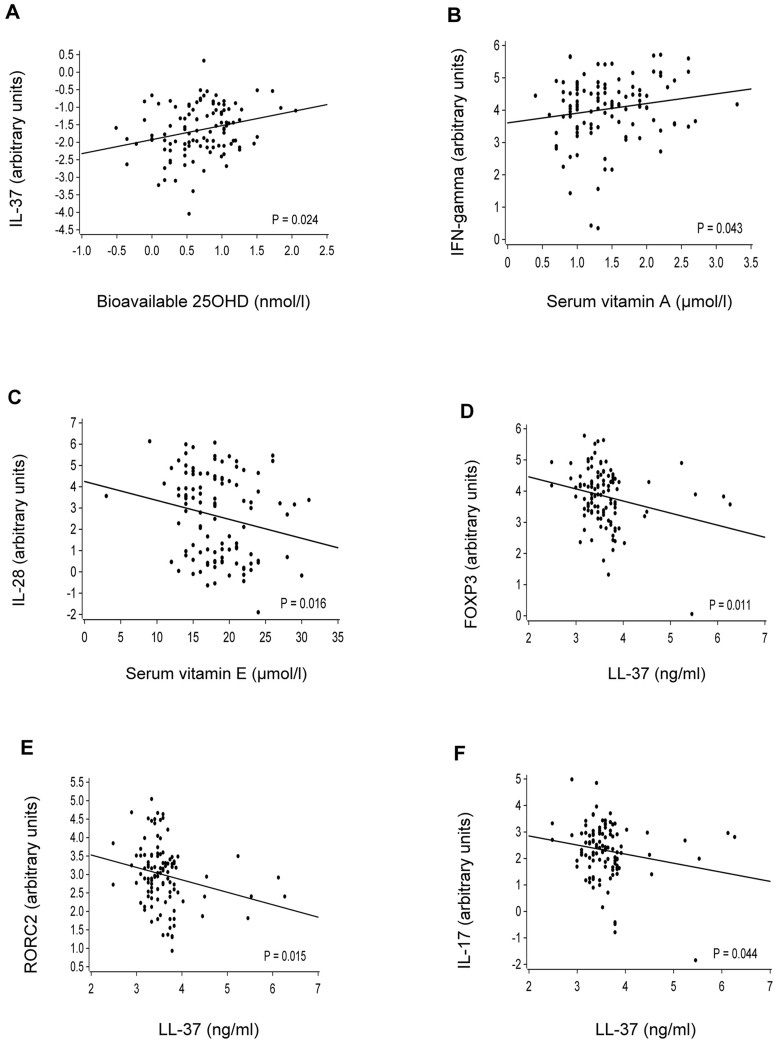
Scatter plots and regression lines between serum (A) bioavailable 25(OH)D and IL-37; (B) vitamin A and IFN-γ, (C) vitamin E and IL-28; (D) LL-37 and FOXP3; (E) LL-37 and RORC2; (F) LL-37 and IL-17. P-values were adjusted to age except in figures (D-F).

In age-adjusted analysis, we observed that higher bioavailable 25(OH)D levels were associated with higher expression levels of newly discovered anti-inflammatory cytokine IL-37 (*P* = 0.024) (Fig 4A) and higher vitamin A levels were associated with higher expression of IFN-γ (*P* = 0.043) (Fig 4B). Higher vitamin E levels were associated with lower IL-28 expression (*P* = 0.016) (Fig 4C). In addition, higher serum antimicrobial peptide LL-37 levels were associated with lower expression of intratonsillar FOXP3 (*P* = 0.011) (Fig 4D), RORC2 (*P* = 0.015) (Fig 4E) and IL-17 (*P* = 0.044) (Fig 4F). No other significant associations were found between serum levels of vitamins and LL-37 and the “immune activation/regulatory” cluster of cytokines and their transcription factors in tonsils. Age, smoking or indication for tonsillectomy did not have modifying effects on the mRNA expression associations.

## Discussion

This study provides new insights into connections between serum levels of vitamins A, D, and E and antimicrobial peptide LL-37 and several important outcomes: allergy, respiratory virus detection and tonsillar immune responses. We found that higher bioavailable 25(OH)D levels were associated with lower prevalence of allergic rhinitis and atopy, higher vitamin E levels with lower prevalence of self-reported allergy, and higher vitamin A showed borderline significance for an association with less respiratory virus detections. In line with this finding, we found that higher vitamin A levels were associated with higher intratonsillar expression of IFN-γ. Also, higher serum bioavailable 25(OH)D levels were associated with higher intratonsillar expression of novel anti-inflammatory cytokine IL-37, which is known to suppress immune responses, regulate T-reg development and induce tolerance [23].

Exacerbations of childhood and adult asthma are often caused by viral infection [24]. It is generally accepted that, low or deficient innate and adaptive immune responses may contribute to the morbidity of viral infections [25]. We show that multiple viruses exist in nasopharynx and tonsils in relatively asymptomatic patients. Less vitamin A was associated with less IFN-γ and tendency for more viral detection, which may partly explain the association previously seen with vitamin A deficiency and asthma exacerbations [1]. The inverse association between vitamin A levels and virus detection in our study is interesting since multiple observational studies have shown that vitamin A deficiency is associated with a higher risk of asthma and wheezing [5–7, 26, 27]. Vitamin A has been shown to enhance Treg activity via FOXP3 and inhibit Th17 development via retinoid orphan receptor γt (RORγt) [28–31], but we did not find any significant association between serum vitamin A levels and intratonsillar antiviral/immunoregulatory gene expression, except for IFN-γ. IFN-γ is a critical molecule in immune system with multiple functions, mostly related to Th1 response against bacterial, viral and fungal infections [32].

Vitamin D has beneficial pleiotropic effects on both the innate and adaptive immune system [33]. High vitamin D levels during maternity are associated with less childhood wheezing [34–37] and vitamin D deficiency appears to contribute to increased susceptibility to infections and wheezing [12, 38, 39]. Prospective studies have shown that vitamin D supplementation reduces the risk of recurrent respiratory infections, virus-induced wheezing and asthma exacerbations [12]. Even though multiple studies suggest that vitamin D has beneficial effects on the immune defense and on allergic disease, the exact mechanisms are not well defined. Our finding of the positive association with higher vitamin D status and higher intratonsillar expression of anti-inflammatory cytokine IL-37 might partly explain some of these results. IL-37 is an anti-inflammatory cytokine which suppresses immune responses and inflammation [23].

Vitamin D is also known to enhance the expression of antimicrobial peptide cathelicidin, often referred in its active form as LL-37 [15]. LL-37 is not only an endogenous antibiotic peptide that destroy bacteria, virus and fungi, but can also act as an immune modulator [40]. We found that higher serum LL-37 levels were associated with lower intratonsillar expression of IL-17 and its transcription factor RORC2, both needed to Th17 cell development [41], as well as lower expression of FOXP3, a transcription factor known to induce Treg cells [41, 42]. In our study cohort, serum LL-37 levels tended to increase with vitamin D levels, but this correlation did not reached statistical significance (S2 Fig). Our patients did not have acute infection at the time of operation and collection of blood samples. It might be that without acute infection, serum LL-37 levels are not elevated. This data may suggest that a critical balance appear to lie between LL-37 expression due to infections and Th17 and Treg cell development.

Vitamin E levels have been shown to associate with Th1 and Th17 development [43]. Our data shows that serum higher vitamin E levels were associated with less self-reported allergy. In agreement with this finding, maternal vitamin E intake during pregnancy has been associated with less wheezing and eczema in children [16, 17]. We found weak or no associations between serum vitamin E levels and the expression of cytokines or transcription factors in tonsils.

Strengths of our study are simultaneous measurement of multiple vitamins and a novel antimicrobial peptide LL-37, comprehensive viral and atopy characterization and complete clinical data of over 100 patients. Statistical analyses were conducted carefully and adjusted for age, smoking and indication for tonsillectomy. According to a previous report from our group, indication for operation, mainly tonsil hypertrophy or recurrently infected tonsils, do not play a role in expression of studied genes [20]. However, we do not have yet any mechanistic data to understand how these regulatory networks crosstalk.

In summary, our study provides new evidence suggesting that vitamin A may have antiviral effects. Also, our study suggests potentially important roles for vitamin D and antimicrobial peptide LL-37 in Th17 and Treg cell regulation and development of allergic disease. Clinically, our study suggests that vitamin D may promote anti-inflammatory mechanisms. Further studies are needed to understand the crosstalk between regulatory networks in allergy and viral infections.

## Supporting information

S1 Fig Correlations between age and (A) serum total vitamin D, (B) serum LL-37. Spearman’s correlations are shown. Regression lines have been added for better visualization.
(TIF)

S2 Fig Correlations between serum LL-37 and (A) total vitamin D, (B) bioavailable vitamin D. Spearman’s correlations are shown. Regression lines have been added for better visualization.
(TIF)

## Abbreviations

25(OH)D: 25-hydroxyvitamin D
AdV: adenovirus
BoV: bocavirus
CT: cycle threshold
CV: coronavirus
EF1α: elongation factor 1α
EV: enteroviruses
Flu: influenza A or B virus
FOXP3: forkhead box protein 3
HPLC: high-performance liquid chromatography
IFN: interferon
IgE: immunoglobulin E
IL: interleukin
MPV: metapneumovirus
PBMC: peripheral blood mononuclear cells
PCR: polymerase chain reaction
PIV: parainfluenza virus types 1–4
RORC2: retinoic acid receptor-related orphan receptor C2
RSV: respiratory syncytial virus
RV: rhinovirus
TGF-β: transforming growth factor β
Th: T helper cell
Treg: T regulatory cell
