# Supplementary figures and images for: The relationship of serum vitamins A, D, E and LL-37 levels with allergic status, tonsillar virus detection and immune response
Source: PLoS One. 2017 Feb 24;12(2):e0172350. doi: 10.1371/journal.pone.0172350 (PMC5325266; doi:10.1371/journal.pone.0172350)

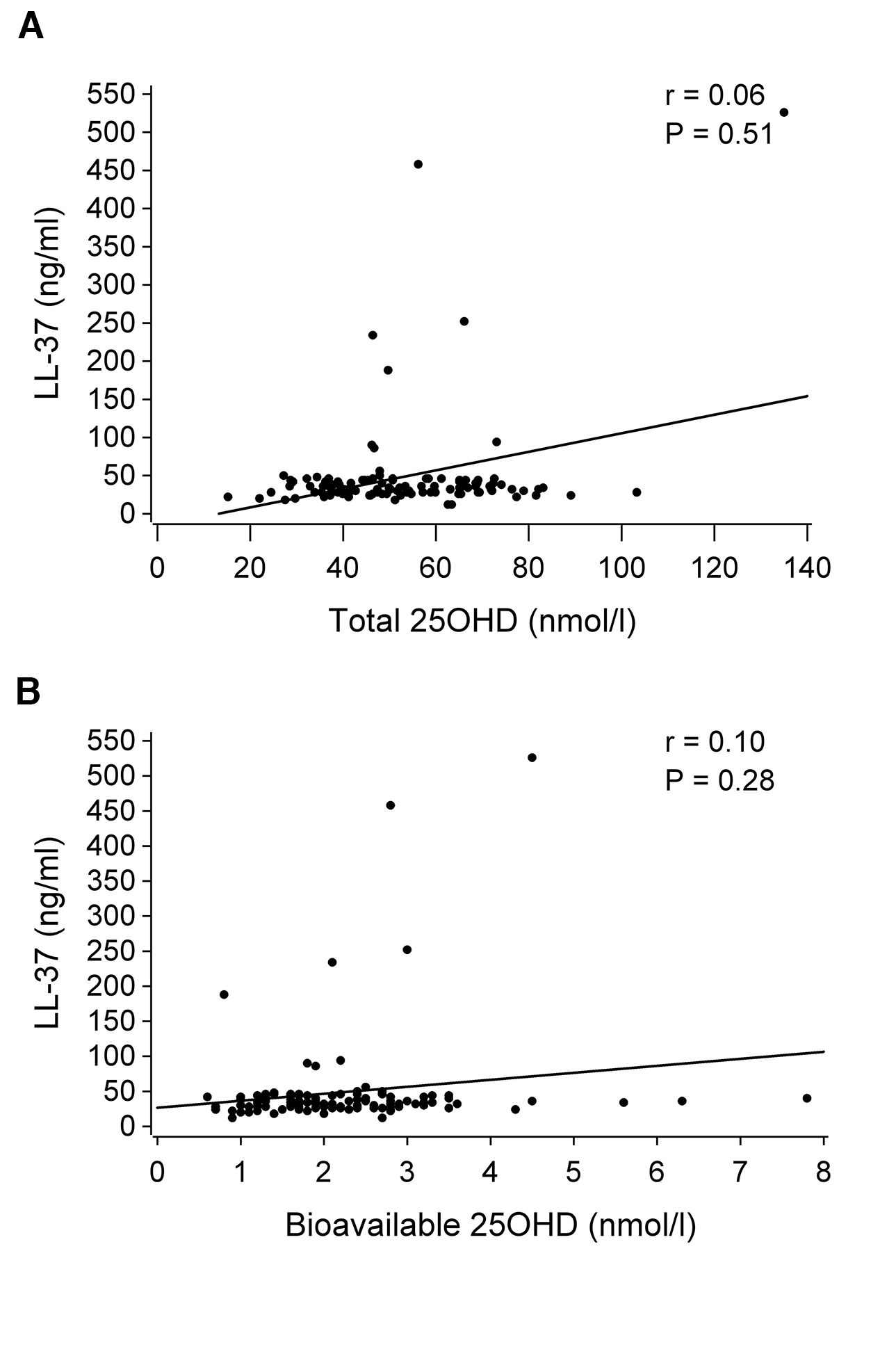

Supplement: S2 Fig — Spearman’s correlations are shown. Regression lines have been added for better visualization. (TIF) [file pone.0172350.s002.tif]
